# Pre-surgical radiologic identification of peri-prosthetic osteolytic lesions around TKRs: a pre-clinical investigation of diagnostic accuracy

**DOI:** 10.1186/1749-799X-3-47

**Published:** 2008-10-03

**Authors:** Timothy P Kurmis, Andrew P Kurmis, David G Campbell, John P Slavotinek

**Affiliations:** 1 Department of Orthopaedic Surgery, Flinders Medical Centre, Bedford Park, South Australia, Australia; 2 School of Medicine, Flinders University, Bedford Park, South Australia, Australia; 3 Wakefield Orthopaedic Clinic, Adelaide, South Australia, Australia; 4 Division of Medical Imaging, Flinders Medical Centre, Bedford Park, South Australia, Australia

## Abstract

**Background:**

Emerging longitudinal data appear to demonstrate an alarming trend towards an increasing prevalence of osteolysis-induced mechanical failure, following total knee replacement (TKR). Even with high-quality multi-plane X-rays, accurate pre-surgical evaluation of osteolytic lesions is often difficult. This is likely to have an impact on surgical management and provides reasonable indication for the development of a model allowing more reliable lesion assessment. The aim of this study, using a simulated cadaver model, was to explore the accuracy of rapid spiral computed tomography (CT) examination in the non-invasive evaluation of peri-prosthetic osteolytic lesions, secondary to TKR, and to compare this to conventional X-ray standards.

**Methods:**

A series of nine volume-occupying defects, simulating osteolytic lesions, were introduced into three human cadaveric knees, adjacent to the TKR implant components. With implants *in situ*, each knee was imaged using a two-stage conventional plain X-ray series and rapid-acquisition spiral CT. A beam-hardening artefact removal algorithm was employed to improve CT image quality.

After random image sorting, 12 radiologists were independently shown the series of plain X-ray images and asked to note the presence, anatomic location and 'size' of osteolytic lesions observed. The same process was repeated separately for review of the CT images. The corresponding X-ray and CT responses were directly compared to elicit any difference in the ability to demonstrate the presence and size of osteolytic lesions.

**Results:**

Access to CT images significantly improved the accuracy of recognition of peri-prosthetic osteolytic lesions when compared to AP and lateral projections alone (*P *= 0.008) and with the addition of bi-planar oblique X-rays (*P *= 0.03). No advantage was obtained in accuracy of identification of such lesions through the introduction of the oblique images when compared with the AP and lateral projections alone (*P = *0.13)

**Conclusion:**

The findings of this study suggest that peri-prosthetic osteolytic lesions can be reliably described non-invasively using a simple, rapid-acquisition CT-based imaging approach. The low sensitivity of conventional X-ray, even with provision of supplementary bi-planar 45° oblique views, suggests a limited role for use *in situ *for TKR implant screening where peri-prosthetic osteolytic lesions are clinically suspected. In contrast, the accuracy of CT evaluation, linked to its procedural ease and widespread availability, may provide a more accurate way of evaluating osteolysis around TKRs, at routine orthopaedic follow up. These findings have direct clinical relevance, as accurate early recognition and classification of such lesions influences the timing and aggressiveness of surgical and non-operative management strategies, and also the nature and appropriateness of planned implant revision or joint-salvaging osteotomy procedures.

## Introduction

Peri-prosthetic osteolytic lesions around orthopaedic implants are a recognised cause of bony matrix instability leading to mechanical failure [1-5]. While several postulates have been suggested to explain this frequently observed phenomenon, the exact mechanism remains controversial [1,3,6-8] and is the subject of current international scrutiny [9]. What does appear to be universally accepted is the need to recognise the onset and progression of osteolytic lesions. This is aimed to be ascertained at the earliest possible point so that appropriate management can provide the best possible clinical and patient outcome [8,10]. To facilitate such practice, there is a need for an accurate and reliable non-invasive technique to allow both lesion identification and morphologic (volumetric) description.

In many countries total knee replacement (TKR) is the most common form of joint replacement [11]. Extensive epidemiological data indicate that the trend towards an increasing incidence of TKR is likely to continue [9]. In a population with an increasing life expectancy [7], there are ever-greater expectations for the preservation of mobility and physical activity [7]. While the vast majority of cases show good clinical outcome and improvement in post-procedural standard-of-life [7], implant failure (through a variety of mechanisms) remains a problematic clinical issue [9]. Particle-induced wear-related bone loss (osteolysis) is a recognised precursor to implant loosening and mechanical instability [8,12]. Osteolysis is often insidious and asymptomatic [10,13,14] until it reaches critical levels, with subsequent implant failure. For this reason, peri-prosthetic osteolysis following TKR has become a significant clinical problem [8,15]. Periodic radiographic surveillance post-joint replacement is often prospectively recommended [8,16,17], especially for young and active recipients [18]. This allows early detection and thus instigation of management pathways [8,18,19], aiming to achieve better long-term patient outcomes.

In the majority of cases, post-surgical or follow up plain film X-rays form the routine basis for assessment of implant positioning, stability and integrity, as well as evaluation of the condition of adjacent bony domains [20-22]. A small number of institutions employ conventional CT-based follow up either as an adjunct to, or *in lieu *of, plain film examinations [10]. However, in most cases, such practice is likely to involve isolated patients on a purely case-by-case basis, commonly with a more pressing secondary indication.

Historically, the use of plain film X-ray examinations as a screening tool for osteolysis, despite multi-angle and multi-projection approaches, has proved unreliable [5,21,23]. Concerns have been raised regarding the inability to accurately delineate the peripheral margins of osteolytic lesions, often resulting in under-estimation of lesion size [10,18,21,23-25] (especially in close proximity to the bone/implant interface). Additionally, they often lack consistency and repeatability in sequential (follow-up) examinations, limiting direct comparability and hence clinical benefit in the accurate monitoring of progressive change [26]. The latter is heavily influenced by subtle variations in patient presentation and radiographic technique (i.e. patient positioning, central beam orientation, exposure parameters, projection series performed and structural superimposition) [18,21,22,27,28]. Although often advocated [10,28], the application of conventional CT for non-invasive osteolytic lesion description, has been limited by poor scan alignment on longitudinal assessment. This has subsequently resulted in inaccurate extrapolation of volume estimates when viewing sectional images. Also, the presence of metal (i.e. implant) in the scan field causes significant image distortion due to beam hardening artefact [5,28-30] and inherently limits the clinical value of obtained images [28,31].

The description of osteolytic lesions and their size around total hip replacements (THR) has been reported previously [5,32,33] and appears to be relatively common [10]. However, there is little evidence in the contemporary literature to suggest that substantial application of such approaches have been extrapolated to other body regions, including the human knee.

There is increasing suggestion that CT-based assessment of peri-prosthetic bone around TKRs may provide a quick, technically simple, highly accurate and reliable form for volume measurement of both discrete pathology and normal anatomy [5,28,30]. Ongoing advancements in CT scanner-based algorithms for the reduction (or amelioration) of metal (i.e. implant) induced beam hardening artefact [5,26,31,34], combined with next generation software-based correction techniques [34], have largely overcome many of the pitfalls previously associated with orthopaedic imaging. These technologies provide a non-invasive imaging modality, which may be inherently suited to analysis of osteolysis in the peri-prosthetic region [5,14,26,30,34].

Given the clinical relevance of accurate description of TKR-associated peri-prosthetic osteolysis, and the lack of evidence indicating previous similar work, the aim of this study was to assess lesion recognition and description using a rapid-acquisition CT-based imaging technique, and to contrast this to standard X-ray examination approaches.

## Materials and methods

Three *ex vivo *cadaver knee specimens were obtained following institutional ethics committee approval. Appropriately sized cementless tibial arthroplasty components (PFC sigma standard, DePuy Orthopedics, Warsaw, Indiana, USA; Genesis 2 tibial component, Smith & Nephew, Memphis, Tennessee, USA; Genesis 1 cementless tibial component, Smith and Nephew, Memphis, Tennessee, USA) were inserted into each specimen by an experienced orthopaedic surgeon, using standard surgical implantation techniques and the provided proprietary equipment.

With implants *in situ*, baseline imaging of each knee (t = 0) was performed using an Aquilion multi-purpose CT scanner (Toshiba Medical Systems, California, USA) and a conventional helical acquisition technique (120 kV, 250 mA, 0.5 sec rotation, 16 × 0.5 mode SFOV 320 mm TCOT recon method). A conventional beam hardening artefact removal algorithm (Boost dynamic 3D artefact reduction filter) was employed at the time of acquisition to improve resultant image quality. CT data were filmed as standard 4 × 6 sheets. Plain film X-rays in the antero-posterior (AP), lateral and paired 45° AP-oblique projections were also obtained using standard (clinical) radiographic imaging techniques.

**Figure 1:**
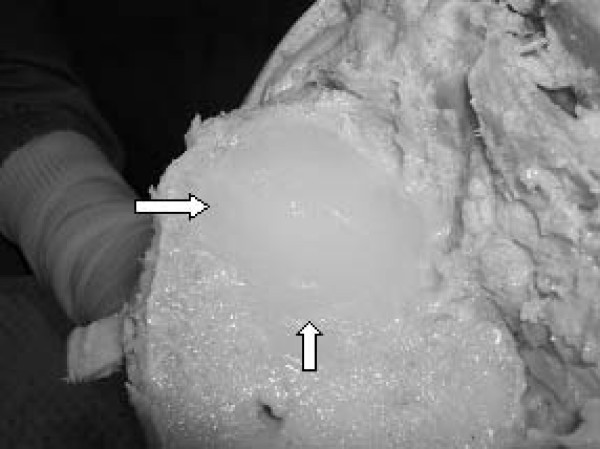
A 'large' tibial osteolytic defect filled with silicon pre-implant insertion.

**Figure 2:**
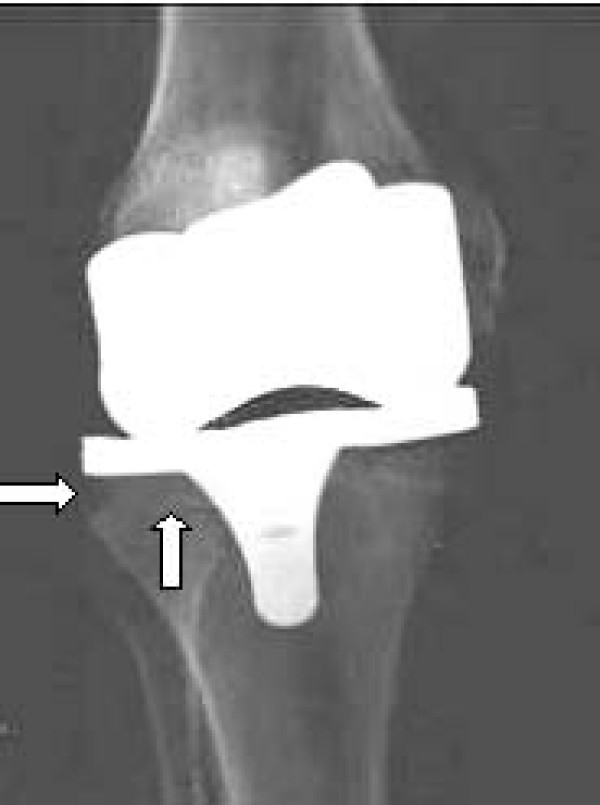
**Antero-posterior plain X-ray of tibial osteolytic defect (large) as shown in Figure **1.

**Figure 3:**
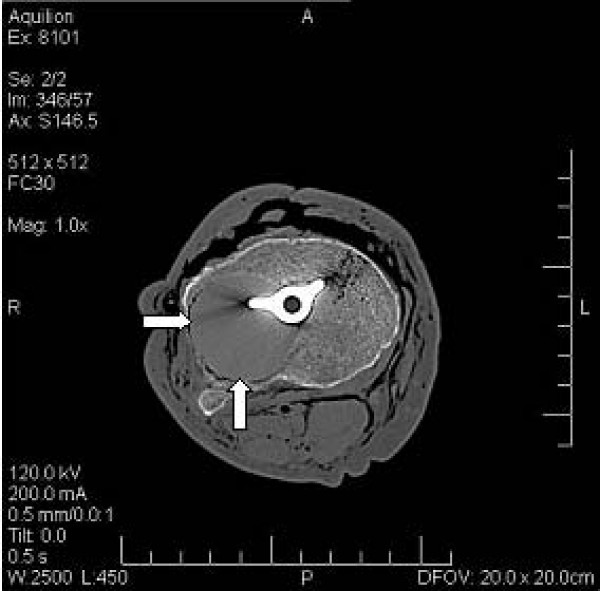
**Axial CT scan of tibial osteolytic defect as shown in Figure** 1.

Post-imaging, the implant components were removed and, in a method similar to that previously described by Nadaud et al. (2004) [8] and Claus et al. (2003 & 2004) [21,34], volume-occupying osteal defects were introduced immediately adjacent to the tibial implant component, to simulate an osteolytic lesion. Lesions were created using a standard acetabular reamer. The resultant negative bone defects were filled with clear, low-density, silicon (Parfix: Selleys Pty Ltd; Padstow, NSW, Australia) to provide a non-osseous tissue density, ameliorating the formation of an intra-substance, air-bone interface during imaging (Figure 1). The implants were re-inserted in anatomical alignment, soft-tissue overlays were again closed, and the knees were subjected to plain film (Figure 2) and CT imaging (Figure 3) under identical parameters as those employed for baseline imaging (t = 1).

The above method was repeated on two further occasions (i.e. t = 2; t = 3), with the production of progressively larger defect sizes. Approximate lesion sizes and anatomical distribution were modelled on prospectively collected data analysing the clinically observed pattern of osteolysis, resultant from polyethylene-related *in vivo *implant wear, as observed at the host institution (unpublished data). The lesion sizes were then classified as either 'small' (t = 1), 'medium' (t = 2) or 'large' (t = 3) to assist in further analysis of data obtained. In total, nine osteal lesions were induced in the three knees resulting in 36 sets of images, including baseline images.

Each image/image series was prospectively allocated a four-digit identification number to ensure donor anonymity and allow image tracking. The code linking the identification number to any held patient data was only made available to the first two authors.

Following completion of a standard observer participation/consent form, lateral and AP X-ray images from each of the four time points (i.e. t = 0, t = 1, t = 2, t = 3), for each of the three knees were shown in random order, using an observer blinded approach, to 12 radiologists (6 registrars, 4 advanced trainees, 2 consultants) independently, who were asked to record whether or not they felt each set of images demonstrated a peri-prosthetic osteolytic lesion and give an approximate estimation of size (mm^3^). Subsequently, the paired 45° oblique plain X-ray views corresponding to each AP/lateral image set was introduced, and the observer asked to repeat the diagnostic process described above.

Finally, without access to the plain X-ray data (or the previously recorded image assessments), and in a random order not corresponding to the presentation sequence used for plain X-ray film evaluation, observers were shown the spiral CT data for each of the four time points, for each of the three knees, using the same criteria as used previously.

Efforts were made to ensure consistency of the viewing conditions for each observer (i.e. environmental noise levels, ambient lighting etc.). Each observer viewed the images in the same sequence (to avoid presentation bias), although this represented a random order with respect to the knee or time point being presented. One member of the research team was present during all image evaluation sessions.

### Statistical methods

Paired *t *testing analysis was used to compare the three imaging methods (i.e. AP/lateral plain film X-rays alone; AP/lateral plain film X-rays plus paired 45° AP-obliques; CT imaging) with regards to the accuracy of lesion identification. Accuracy was calculated as a percentage, through correct identification of lesions based on the known lesion sizes and sites as per surgical insertion. All statistical functions were performed using the StatView (Abacus Concepts, U.S.A.) data analysis software.

## Results

A total of 12 independent observers were available for study-related image assessment. For each of the lesion sizes, the mean volume was calculated using the mass of each lesion and the density as supplied by the manufacturer of the silicon (small 0.8 cm^3^; medium 2.6 cm^3^, large 10.5 cm^3^). Mean accuracy in the identification of osteolytic lesions for all volumes was 52.1%, with access to plain film AP/lateral X-rays alone. In comparison, observer accuracy increased marginally to 56.3% with the added availability of paired 45° AP-obliques, but rose to 71.5% with provision of CT data.

Analysis was performed using paired *t *testing to compare accuracy in lesion identification and description of the lesion (small, medium or large). Statistically significant differences were observed in accuracy in diagnosis when comparing CT and AP/lateral (*P *= 0.08) and CT versus AP/lateral and oblique X-rays (*P *= 0.03). However, there was no advantage demonstrated through the introduction of oblique X-rays in comparison to AP/lateral images alone (*P *= 0.13). Further analysis was performed for accuracy of diagnosis of lesions based on their size (small, medium or large). CT was shown to be superior in identification of 'large' lesions when compared to AP/lateral X-ray (*P *= 0.03), however, no difference was observed between diagnostic accuracy when CT was compared to paired oblique X-rays (*P *= 0.34) nor AP/lateral compared with paired oblique images (*P *= 0.06). For those lesions deemed to be 'medium', CT was superior to AP/Lateral X-rays (*P = *0.02) and paired oblique X-rays (*P = *0.01). Again, there was no advantage with the addition of paired oblique X-rays compared with standard AP/lateral projections (*P *> 0.99). When comparing imaging modalities for those lesions deemed to be 'small', once again CT was shown to be superior to AP/lateral projections (*P = *0.004). However, there was no statistical significance demonstrated through the use of CT versus the standard projection and paired oblique combination (*P = *0.78). Paired oblique and AP/lateral combination X-rays was shown to be superior to AP/lateral projections alone (*P = *0.05) in the identification of 'small' lesions.

## Discussion

The purpose of this study was to determine the accuracy of conventional spiral CT for identification of peri-prosthetic bony defect lesions around TKRs. Even with access to high-quality multi-plane X-ray images, pre-surgical assessment of the size of osteolytic lesions is difficult to accurately ascertain. This is likely to have an impact on surgical management practice and provides reasonable indication for the development of a model which will allow more accurate and reliable lesion assessment.

Our results indicate that radiologists are more accurate in the identification of osteolytic lesions around TKRs when using CT images versus plain AP/lateral X-ray with or without the addition of paired 45° oblique X-rays. When comparing imaging modalities/projections according to the size of the lesion, our results have shown that there may be no difference in the accuracy of identification of small lesions between CT and the combination of AP/lateral and paired oblique X-rays. While the main focus of the present study, one may suggest that this result may have been obtained as a consequence of a small cohort size, perhaps having been too small to show a statistical difference. Future research may be needed to investigate the accuracy of CT for the identification of small lesions alone, using a larger cohort.

At the other end of the lesion scale (large), there was no demonstrated advantage in using CT over the combination of AP/lateral and paired oblique X-rays. Given the substantive size of the lesions, this perhaps is not surprising as one may postulate an osteolytic lesion of such magnitude would be catastrophic for a patient and clinically symptomatic some time earlier, and thus identified earlier.

Anecdotally, more experienced observers are thought to be more capable of identification of such lesions, however the number of observers in our study was not sufficient to provide strong statistical evidence to support this. Therefore, another potential area for future research may involve comparison of the abilities of junior and senior radiologists to identify such lesions. However, we do believe that the range of experience of observers utilized here is representative of clinical expertise present in a general tertiary referral medical facility.

In acknowledging the potential limitations of our work, although we attempted to best replicate *in vivo *conditions using our controlled cadaver model, as would be expected there was a lack of tissue responsiveness to insertion and implant/bone interactions, in contrast to that seen in living patients post-TKR. This may have subsequently influenced the appearance and development of osteolytic lesions resulting in subtle differences to our model. However, we suggest that our study methods allowed for a controlled, highly reproducible tissue environment, appropriate for pre-clinical investigation.

Additionally, the homogeneous nature of the silicon may have not uncategorically reflected the imaging presentation of 'generalised' peri-prosthetic osteolytic lesions, as observed clinically. As a preliminary, pre-clinical study, it was not the intent of this investigation to achieve definitive clinical realism, rather to provide a platform facilitating initial determination of value (or lack of) in the use of rapid acquisition CT technique in the semi-quantitative evaluation of osteolytic lesions. It is hoped that the findings presented here will provide scientifically rigorous evidence to support future *in vivo *analyses in active patient populations.

We also acknowledge that our study only investigated the identification of osteolytic lesions around the tibial component of a TKR. Extension of this premise to other implant types, including the curved femoral component of a TKR, provides an avenue for further targeted research.

Our data set of 432 discrete diagnoses (i.e. identification of 27 lesions, plus 9 'lesion-free' images, by 12 observers) provides some degree of confidence in the external validity of findings, although further prospective clinical trials are required to ascertain the true value of CT-based approaches in the screening of *in situ *TKRs in the clinical setting.

Taking the above into consideration and the wide availability, relatively low cost and ease for patients (i.e. no significant moving) of CT scanning, combined with the ability of direct or post-acquisition image reformatting, our findings indicate a more accurate alternative to the previously accepted value of using 'routine' plain X-ray examination of *in situ *TKRs, in the tertiary care setting. Our results suggest that radiologists are more accurate in diagnosing osteolytic lesions around TKRs with CT scanning and such an approach may be considered a more appropriate first-line investigation method, especially where the clinical suspicion of an osteolytic lesion is high.

## Conclusion

The findings of this study indicate the presence of peri-prosthetic osteolytic lesions around TKRs can be accurately described non-invasively in an *in situ *setting, using conventional spiral CT. Additionally, we believe that we have shown that plain X-ray examination of TKR-associated osteolytic lesions may not be the most appropriate imaging modality for early diagnosis. Also, our findings suggest that the addition of paired oblique X-rays to standard AP/lateral projections offer no significant benefit in diagnosis and may represent unnecessary effort and radiation exposure. These findings may be of clinical benefit in influencing the timing and aggressiveness of surgical and non-operative patient management strategies and in determining the appropriateness and nature of planned implant revision or salvaging osteotomy procedures.

## Competing interests

The authors declare that they have no competing interests.

## Authors' contributions

All authors read and approved the final manuscript.

## Funding recognition

No funding was utilised for the completion of this project.
